# Prognostic significance of urothelial carcinoma with divergent differentiation in upper urinary tract after radical nephroureterectomy without metastatic diseases: A retrospective cohort study

**DOI:** 10.1097/MD.0000000000006945

**Published:** 2017-05-26

**Authors:** Chuan Qin, En-Li Liang, Zhi-Yong Du, Xiao-Yu Qiu, Gang Tang, Fei-Ran Chen, Bo Zhang, Da-Wei Tian, Hai-Long Hu, Chang-Li Wu

**Affiliations:** a Department of Urology; b Tianjin Key Laboratory of Urology, Tianjin Institute of Urology, the Second Hospital of Tianjin Medical University; c College of Management and Economics, Tianjin University, Tianjin, China.

**Keywords:** divergent differentiation, prognosis, radical nephroureterectomy, tumor location, upper urinary tract urothelial carcinoma

## Abstract

To evaluate the impact of urothelial carcinoma with divergent differentiation (UCDD) on the prognosis of patients for primary upper urinary tract urothelial carcinoma (UTUC) with pN0/x status treated with radical nephroureterectomy (RNU) and to evaluate the prognostic value of UCDD in different tumor locations (renal pelvis and ureter).

Data from a total of 346 patients with UTUC who received RNU between January 2012 and March 2016 in the institution were retrospectively analyzed. Clinicopathological features and prognostic factors age, sex, complaint, height, weight, blood pressure, tumor grade, stage, smoking status, history of adjuvant chemotherapy, tumor location, history of bladder cancer, tumor necrosis, degree of hydronephrosis, tumor size, tumor focality, and preoperative anemia were compared between patients with pure UTUC and patients with UCDD. The endpoints were cancer-specific survival (CSS), overall survival (OS), and intraluminal recurrence-free survival (IRFS).

Overall, divergent differentiation was present in 50 patients (14.5%). UCDD was related to different tumor location (*P* = .01), smoking (*P* = .04), higher body mass index (*P* = .02), and advanced tumor grade (*P* = .01). By Kaplan–Meier analysis, UCDD was found to be significantly correlated with worse IRFS, CSS, and OS (all *P* < .01). Multivariate analysis demonstrated that UCDD was an independent predictor of IRFS (*P* < .01), CSS (*P* = .01), and OS (*P* = .01). However, 40 patients died for various reasons and the 5-year OS rates were 91.9% in UCDD− group and 68.0% in UCDD+ group, respectively. In patients with ureteral tumors, UCDD was the significant predictor for IRFS, CSS, and OS. However, the prognostic value of UCDD was not observed in pyelocaliceal tumors.

The presence of divergent differentiation is associated with inferior survival. UCDD may identify patients at high risks for poor prognosis especially in patients with ureteral tumors. As a result, more attention and follow-up should be given to patients with ureteric urothelial carcinoma.

## 1 Introduction

Upper urinary tract urothelial carcinoma (UTUC) is an uncommon but potentially fatal disease, which accounts for approximately 5% of all urothelial malignancies, including renal pelvicalyceal and ureteric urothelial carcinoma.^[1]^ Although patients with UTUC generally receive gold standard treatment, that is, radical nephroureterectomy (RNU) with excision of the bladder cuff, UTUC remains a malignancy with a high potential for local and distant recurrence, especially in patients with advanced disease.^[2]^

Because of the clinical features of UTUC, comprehensive recognition of potential prognostic factors is important to improve therapies. To date, many studies have been conducted to identify the significant prognostic factors of UTUC.^[3,4]^ Tumor stage and grade have often been regarded as the basic prognostic predictors in such cases clinically. Other variables, including lymphovascular invasion (LVI), multifocality, tumor size, and lymph node invasion have also been reported as significantly relevant factors.^[5,6]^ It is generally known pure UTUC accounts for most tumors. However, urothelial carcinoma with divergent differentiation (UCDD), aberrant histological differentiation, is a phenomenon that is well recognized by pathologists.^[7]^ In previous studies, a retrospectively study conducted by Shibing et al^[8]^ demonstrated that UCDD was an independent prognostic factor for cancer-specific survival (CSS), DFS, and overall survival (OS) in patients with UTUC on univariate and multivariate analysis. Moreover, the presence of divergent differentiation in UTUC has been reported to be a significant predictor of prognosis in Japanese populations.^[9]^ However, Rink et al^[10]^ found that UCDD in UTUC was related to poor prognosis on univariate analysis but not on multivariate analysis. Thus, further studies are warranted before UCDD is included in risk prediction tools.

The aim of the study was to estimate the effect of the presence and extent of divergent differentiation on oncological outcomes in patients after RNU in a Chinese population. In addition, the respective influences of UCDD in pyelocaliceal and ureteral tumors were also investigated.

## 2 Patients and methods

### 2.1 Study population

The present study was conducted upon approval from the Institutional Review Board of the Second Hospital of Tianjin Medical University. A total of 517 patients who underwent RNU for UTUC with intent to cure at the hospital from January 2012 to March 2016 were selected for the retrospective analysis. However, 161 patients were excluded from the study because of missing data such as medical reports (n = 45), loss of follow-up (n = 50), lymph node metastasis (n = 39), or conservative surgery, such as segmental ureterectomy and endourological resection of tumor (n = 22). In addition, 10 patients with pure nonurothelial carcinoma were also excluded, and 5 patients with distant metastases were also excluded from the study. The inclusion criteria of clinically disease included pathological stage Ta–T4 without lymph node involvement, and complete surgical resection without positive margins. Finally, 346 patients without distant metastasis comprised the current study cohort. No patients received neoadjuvant chemotherapy and experienced confirmed lymph node metastasis before surgery.

Clinical and pathological information were retrieved from patient charts and electronic medical records. Parameters including age, sex, complaint, height, weight, blood pressure, tumor grade, stage, smoking status, history of adjuvant chemotherapy, tumor location, history of bladder cancer, tumor necrosis, degree of hydronephrosis, UCDD, tumor size, tumor focality, and preoperative anemia were recorded. However, patients with suspicious enlarged lymph nodes on preoperative radiology or with intraoperatively abnormal observations received regional lymphadenectomy. The extent and number of lymphadenectomies performed were determined by the surgeon.

### 2.2 Pathological evaluation

All RNU specimens were processed by genitourinary pathologists at the hospital based on standard procedures. Tumor stage was determined according to the 2010 American Joint Committee on Cancer TNM staging system.^[11]^ Tis, Ta, and T1 tumors were considered as low-stage UTUC, and accordingly, T2, T3, and T4 tumors were grouped into high-stage UTUC. Tumor grading was considered under the 2004 World Health Organization grading system, which is applied in our hospital. Patients who had noninvasive papillary urothelial neoplasm with low malignant potential were regarded as having low-grade papillary urothelial cancer. Tumor location was defined as either renal pelvis or ureter based on dominant tumor features, in sequential order of stage, grade, and size.^[6]^ Tumor size was defined as the maximum diameter of the tumor.^[12]^ History of bladder cancer was defined as concomitant or previous bladder tumors. Patients were classified as demonstrating variant UTUC histology if they presented with UTUC combined with any variant histology. Most of UCDD was identified in combination with immunohistochemical staining, and final diagnoses for unusual and problematic slides were achieved by collective consultation with detailed medical records.

### 2.3 Postoperative follow-up

Follow-up was performed every 3 to 4 months in the 1st year after surgery, semiannually for the 2nd and 3rd year, and annually thereafter, or as clinically indicated. Follow-up included physical examination, blood laboratory tests, chest radiography, urinary cytology, and excretory urography of the contralateral upper urinary tract. Bone scan, chest CT, abdomen CT, or MRI was performed when clinically indicated following institutional guidelines. Intraluminal recurrence was defined as recurrence of tumor in the bladder or contralateral upper urinary tract. The cause of death was identified by physicians via chart reviews or death certificates. Most patients who had advanced UTUC died of widely disseminated metastases. The latest follow-up date was May 1, 2016.

### 2.4 Statistical analysis

The chi-squared test and Student *t* test were used to evaluate the association between categorical and continuous variables, respectively. The characteristics between pyelocaliceal and ureteral tumors were also analyzed. The Kaplan–Meier method was used to estimate the impact of UCDD on survival and intraluminal recurrence. Survival curves were compared using the log-rank test. The potential prognostic factors containing age, sex, complaint, height, weight, blood pressure, tumor grade, stage, smoking status, history of adjuvant chemotherapy, tumor location, history of bladder cancer, tumor necrosis, degree of hydronephrosis, UCDD, tumor size, tumor focality, and preoperative anemia were established by univariate analysis, and only the significant factors were entered into multivariate Cox proportional hazard regression models. Hazard ratios with 95% CIs from the Cox model are used, and *P* < .05 was considered to represent statistical significance. Statistical analyses were performed using SPSS version 19.0 (IBM Corp., Armonk, NY).

## 3 Results

### 3.1 Clinical characteristics

Our study population, consisting of 206 (59.6%) men and 140 (40.4%) women, were divided into 2 groups: UCDD (n = 50, 14.5%) and pure UTUC (n = 296, 85.5%). The demographic and clinicopathological features of 2 groups are shown in Table 1. According to our data, the mean age of the patients was 66.61 ± 9.897 years and the median follow-up was 21 months (range, 1–56 months; interquartile range [IQR], 10–36 months). The prevalence of UCDD was significantly higher in patients with tumor located in renal pelvis (*P* = .01), smoking (*P* = .04), higher body mass index (*P* = .02), and advanced tumor grade (*P* = .01). All clinicopathologic factors between patients with tumors in renal pelvis and ureter were presented in Table 2. Besides, associations between variant histological components and clinicopathological features are presented in Table 3. Squamous cell differentiation was the most common variant UTUC histology (7.5%), followed by glandular differentiation (2.0%) and multiple variant differentiation (2.0%).

**Table 1:**
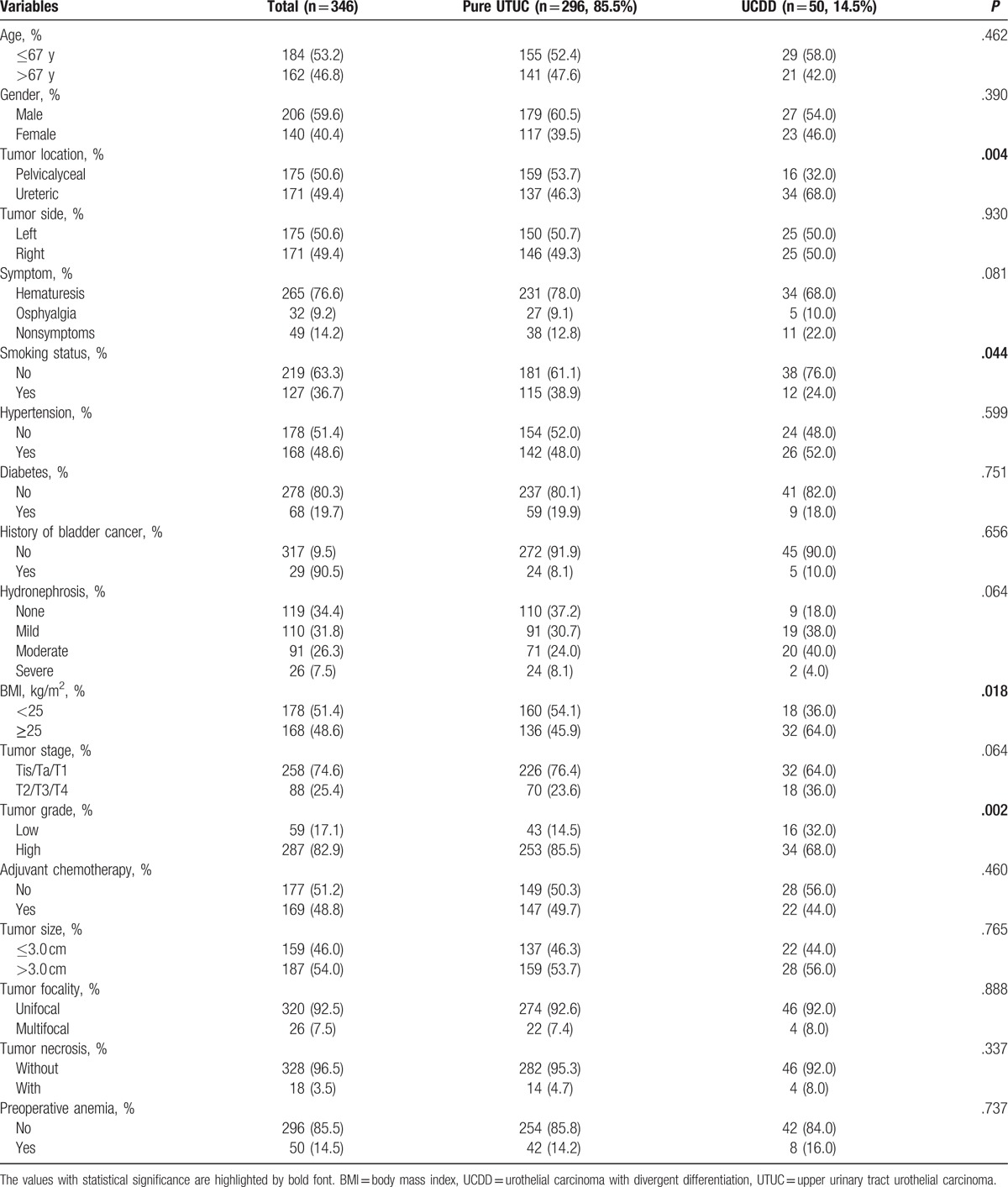
Demographics and clinicopathological characteristics of 346 patients with urinary tract urothelial carcinoma.

**Table 2:**
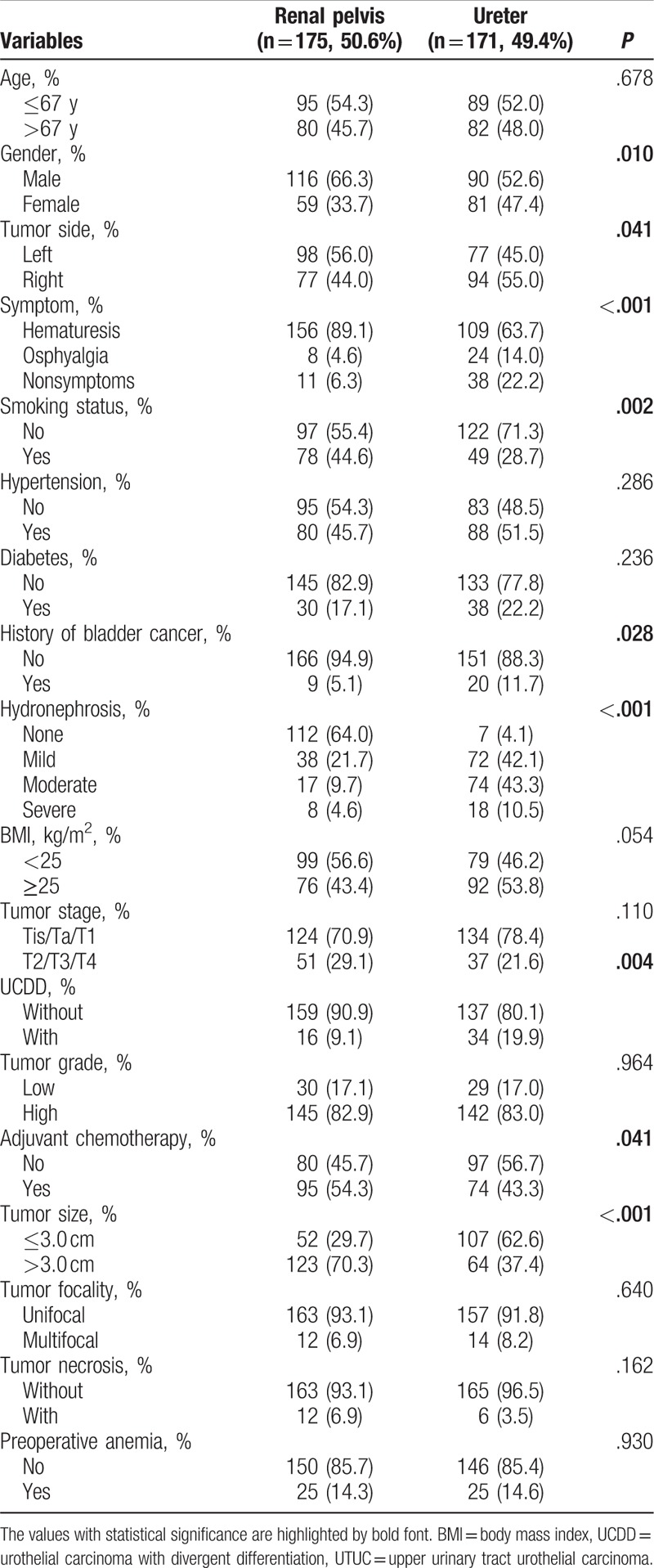
Demographics and clinicopathologic characteristics of 346 patients with UTUC according to tumor location.

**Table 3:**
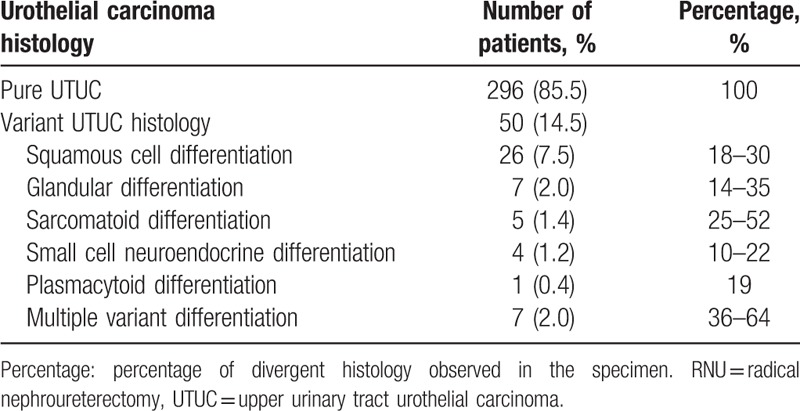
Frequency of urothelial carcinoma with divergent differentiation in 346 patients treated with RNU.

### 3.2 Oncological outcome

**Figure 1:**
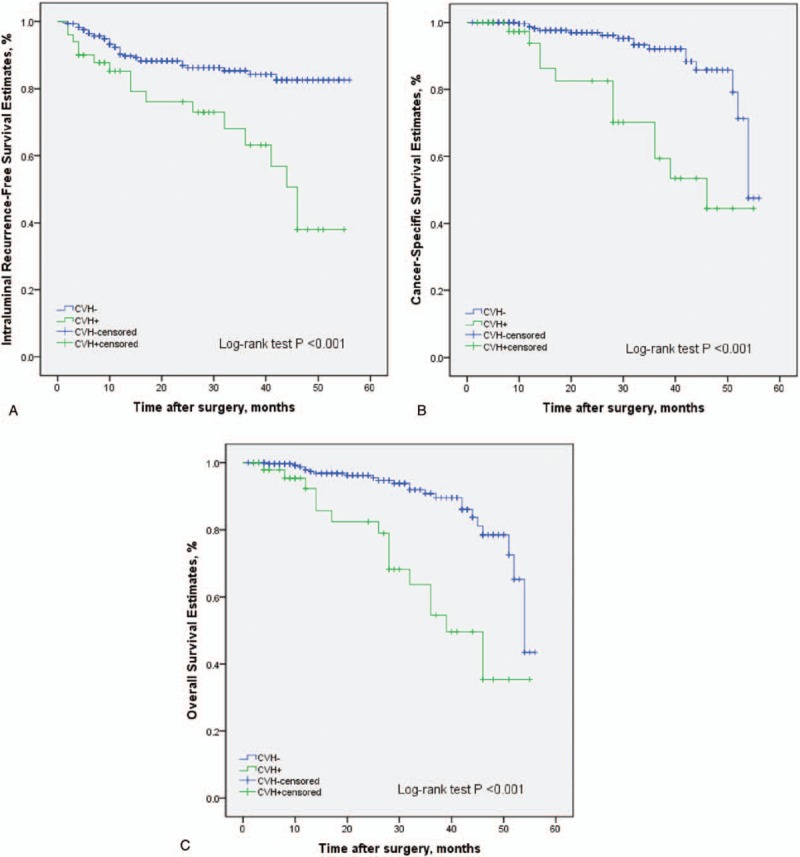
Kaplan–Meier curves for IRFS (A), CSS (B), and OS (C) stratified according to UCDD in 346 patients following RNU of UTUC. CSS = cancer-specific survival, IRFS = intraluminal recurrence-free survival, OS = overall survival, RNU = radical nephroureterectomy, UCDD = urothelial carcinoma with divergent differentiation, UTUC = upper urinary tract urothelial carcinoma.

During the follow-up, 52 patients (15.0%) had an intraluminal recurrence in bladder or contralateral upper urinary tract. The 5-year CSS rate (SD) was 93.9% in the group of pure UTUC and 76.0% in the group of UCDD. Overall, 40 patients died for various reasons and the 5-year OS rates were 91.9% in UCDD− group and 68.0% in UCDD+ group, respectively. Patients with UCDD showed an apparently worse intraluminal recurrence-free survival (IRFS), CSS, and OS than those without UCDD (all *P* < .01, Fig. 1A–C). Univariate and multivariate analysis revealed that UCDD, advanced tumor stage, and grade were independent predictors of adverse IRFS, CSS, and OS (all *P* < .05). Concerning IRFS, UTUC with history of bladder tumor was the most important factor to predict intraluminal recurrence (*P* = .03). In addition, adjuvant chemotherapy in our study may improve outcomes of OS in patients with UTUC after RNU (*P* = .01). The results of univariate and multivariate analyses for prognosis are shown in Tables 4–6.

**Table 4:**
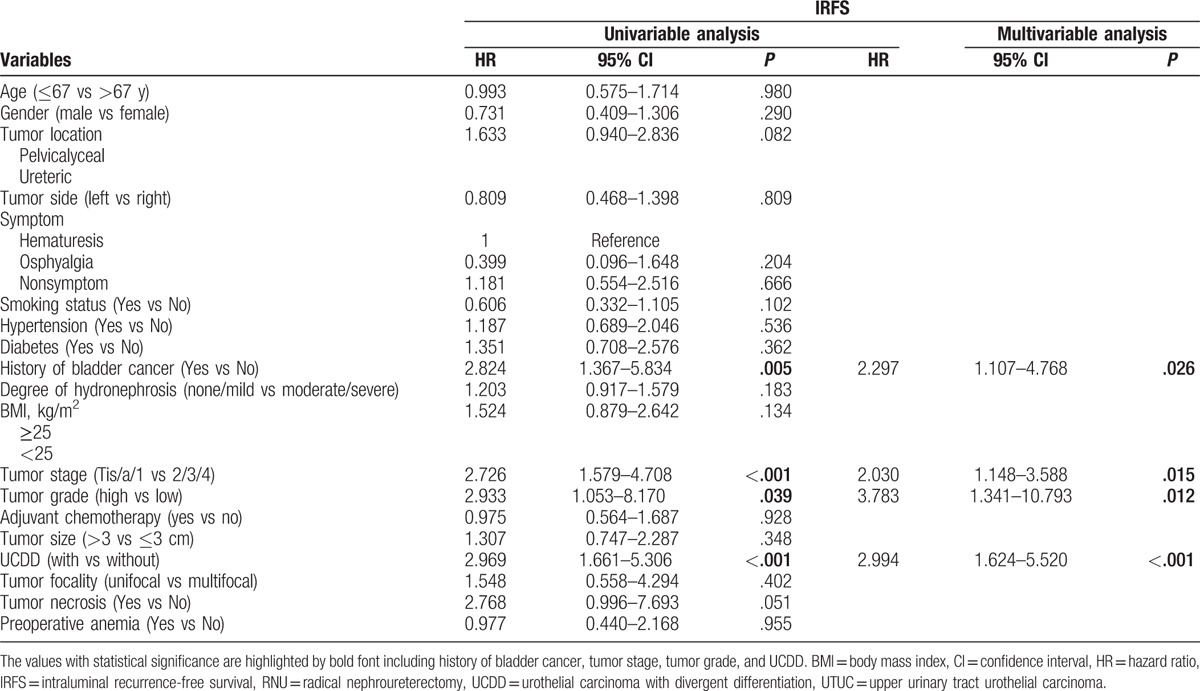
Univariate and multivariate analyses predicting IRFS in 346 patients with UTUC (pN0/X status) after RNU.

**Table 5:**
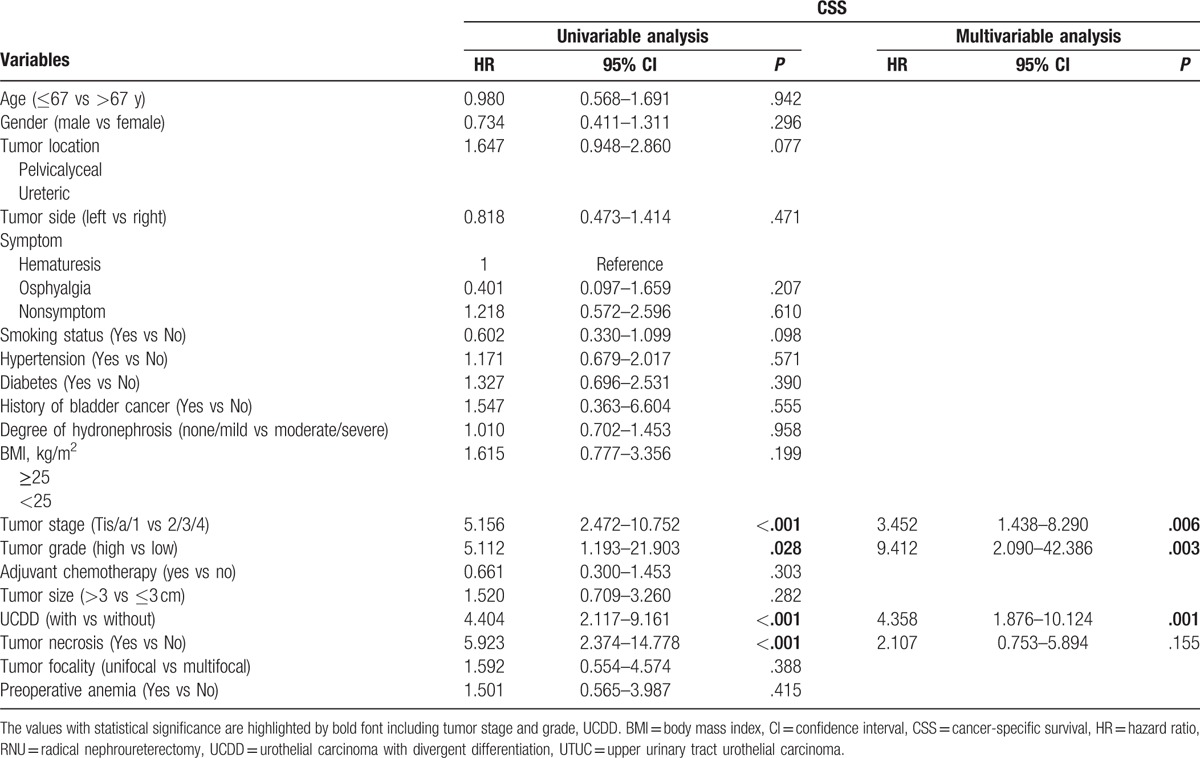
Univariate and multivariate analyses predicting CSS in 346 patients with UTUC (pN0/X status) after RNU.

**Table 6:**
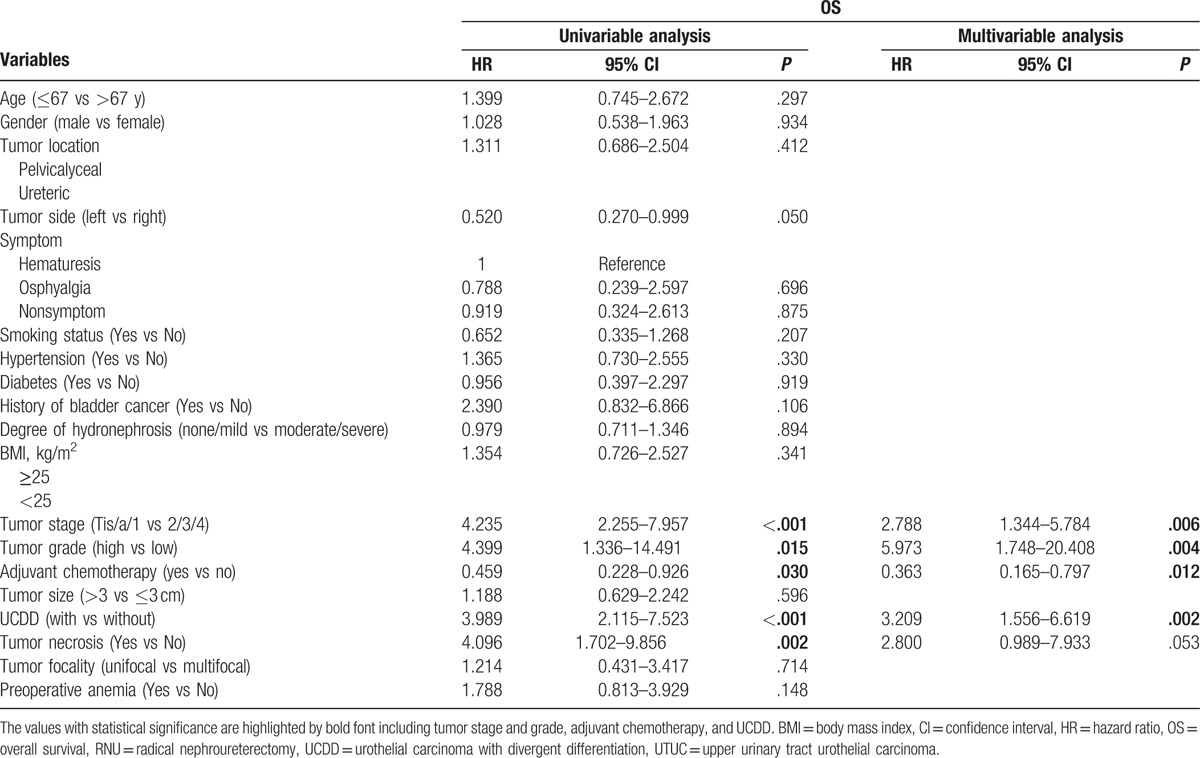
Univariate and multivariate analyses predicting OS in 346 patients with UTUC (pN0/X status) after RNU.

### 3.3 Effect of UCDD in different tumor locations

**Figure 2:**
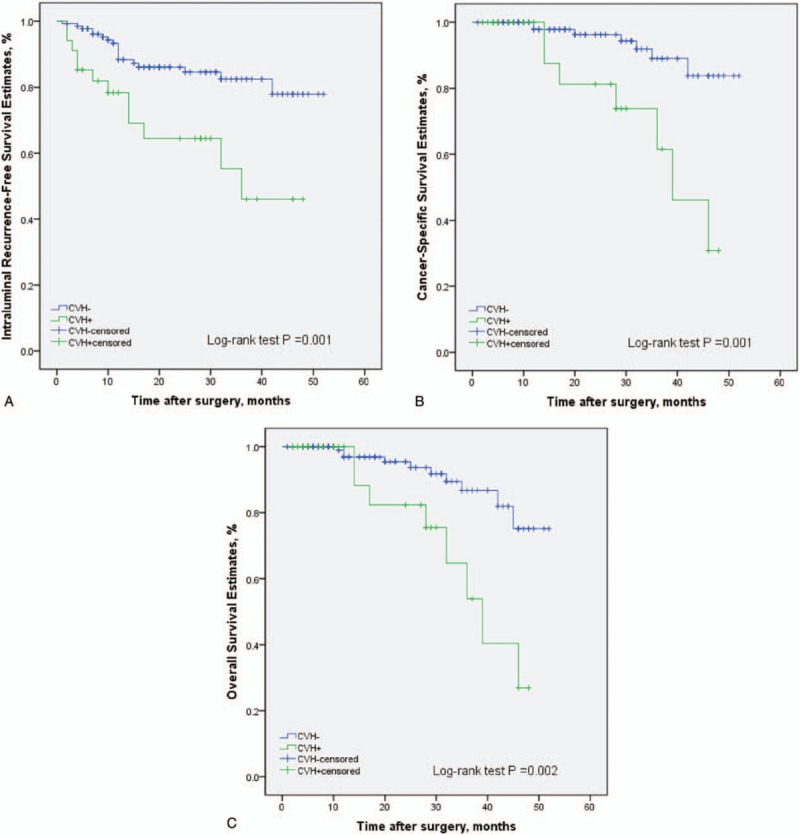
Kaplan–Meier curves for IRFS (A), CSS (B), and OS (C) stratified according to UCDD in patients with ureteral tumors following RNU. CSS = cancer-specific survival, IRFS = intraluminal recurrence-free survival, OS = overall survival, RNU = radical nephroureterectomy, UCDD = urothelial carcinoma with divergent differentiation.

In the subgroup of patients with ureteral tumors, UCDD had a significantly negative impact on IRFS, CSS, and OS (*P* = .01, *P* = .001, and *P* = .01 respectively, Fig. 2A–C). On univariate and multivariate analysis, variant UTUC histology, advanced tumor stage were independent prognostic factors for IRFS, CSS, and OS (all *P* < .05, Tables 7–9). Tumor necrosis predicted a worse prognosis on IRFS (*P* = .02 Table 7). However, the prognostic value of UCDD was not observed in pyelocaliceal tumors. UCDD was not an independent predictor for IRFS, OS on multivariate analysis in pyelocaliceal tumors. There were no significant differences in OS and IRFS between UCDD and pure UTUC in patients with pyelocaliceal tumors (data not shown).

**Table 7:**
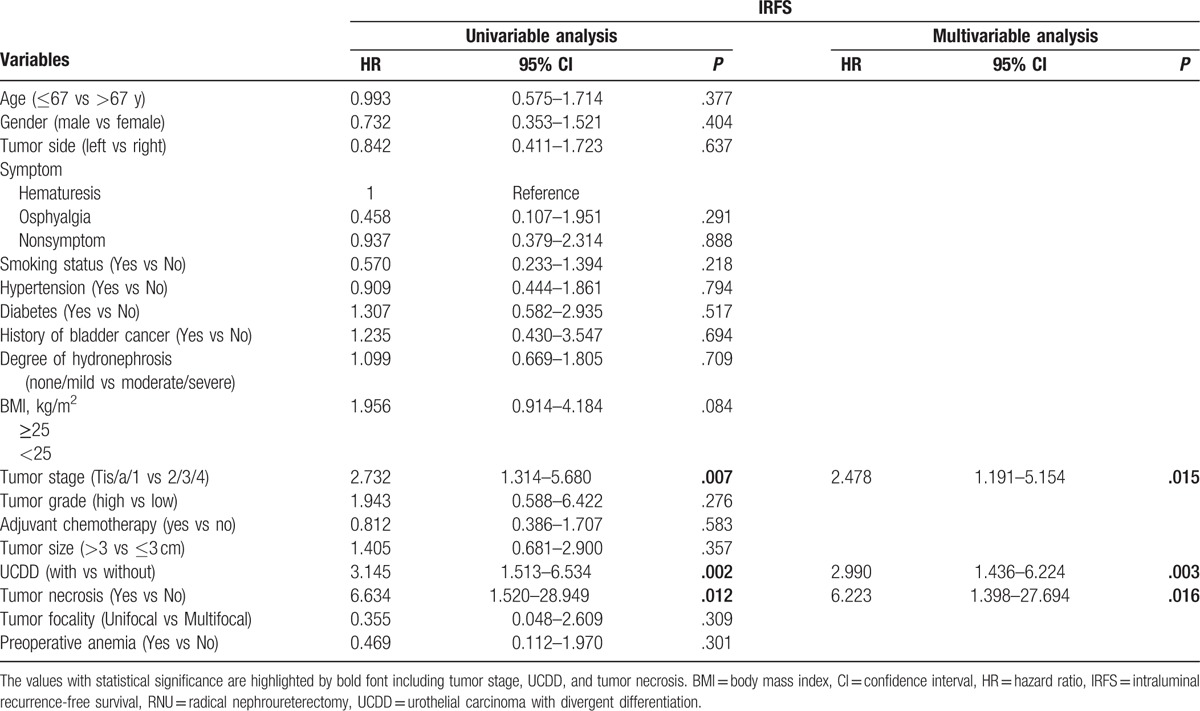
Univariate and multivariate analyses predicting IRFS in patients with ureteral tumor (pN0/X status) after RNU.

**Table 8:**
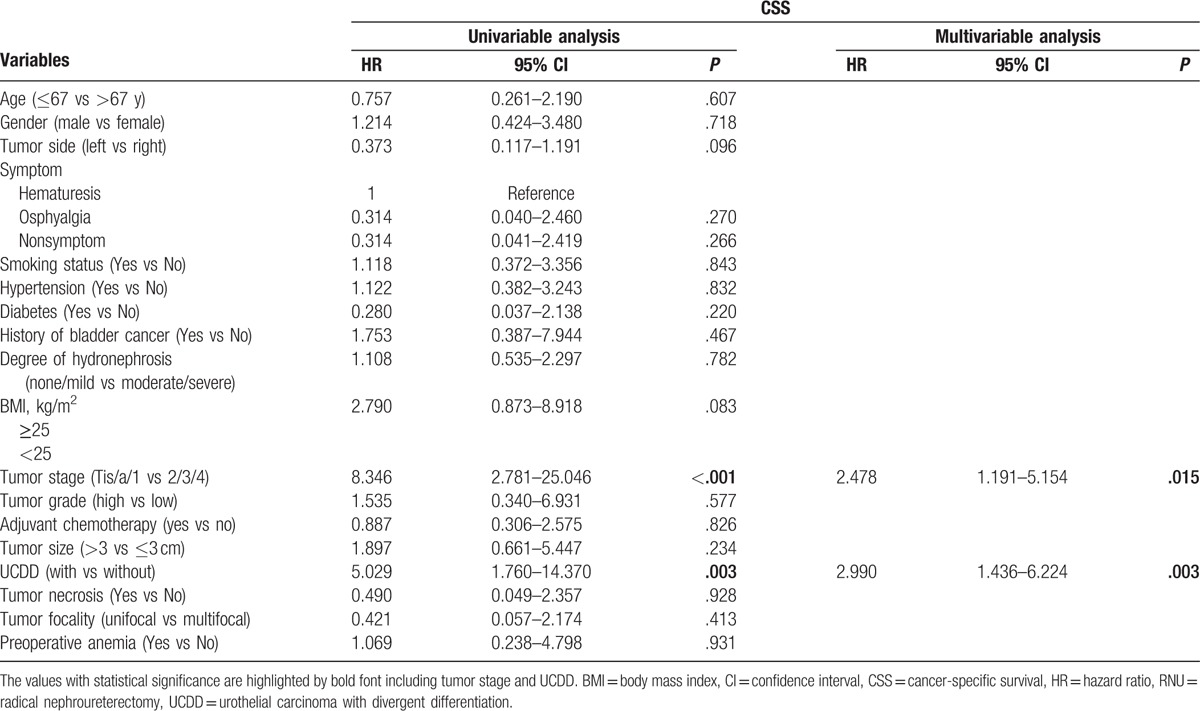
Univariate and multivariate analyses predicting CSS in patients with ureteral tumor (pN0/X status) after RNU.

**Table 9:**
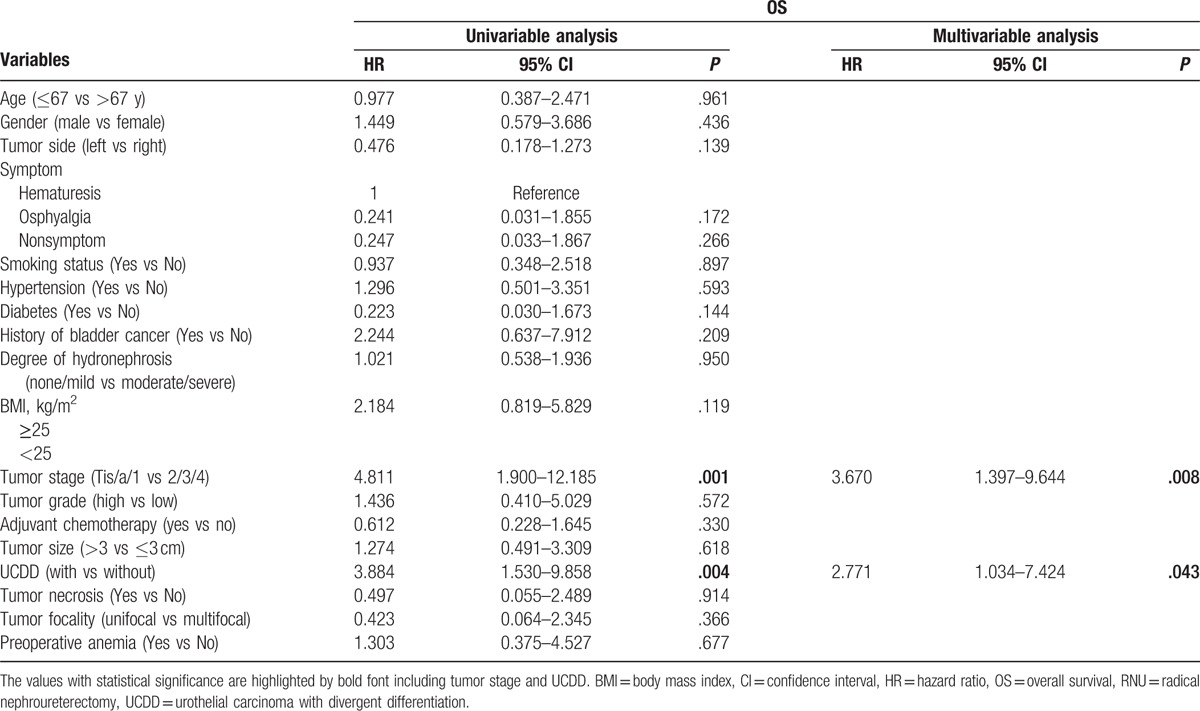
Univariate and multivariate analyses predicting OS in patients with ureteral tumor (pN0/X status) after RNU.

### 3.4 Pathology and immunohistochemistry

**Figure 3:**
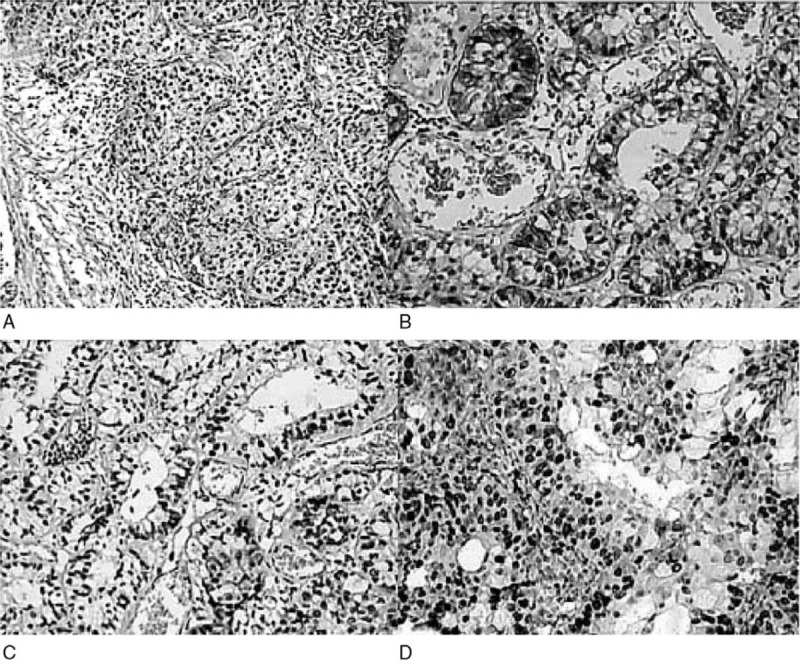
Stained sections in H&E were used to evaluate the presence of divergent differentiation (A) 3AIHC staining of CKp (B), and P63 (C), Ki67 (D) were then performed.

Stained sections in H&E were used to evaluate the presence of divergent differentiation (Fig. 3A), IHC staining of CKp (Fig. 3B), P63 (Fig. 3C), and Ki67 (Fig. 3D) were then performed. IHC stain in these cases was positive for CKp, P63, and Ki67.

## 4 Discussion

UTUC is relatively rare and the prognosis is worse than that of bladder cancer. Thus, identifying prognostic factors to predict a group at high risk of UTUC is crucial to facilitate individualized therapy and proper surveillance protocol, especially those with clinically pN0/x status disease. To date, tumor stage and grade have routinely been used to predict disease prognosis in patients with UTUC.^[13]^ Also, the role of LVI in UTUC has been extensively discussed in recent years.^[14,15]^ In the present study, we retrospectively analyzed the data on 346 patients with UTUC treated with RNU at our institution. We not only confirmed that UCDD was an independent prognostic factor for CSS, IRFS, and OS on both univariate and multivariate analysis among patients with UTUC treated with RNU in this cohort. Furthermore, we found that UCDD is a more essential factor in ureteral tumors than in pyelocaliceal ones.

In the study, we reported that UCDD was present in 14.5% of UTUC specimens, which is similar to the rate of 12% reported by Sakano et al.^[9]^ The patients with UCDD present with more aggressive tumor biological features compared to those with pure UTUC. On the basis of previous reports, some researchers found an association of variant histology with adverse clinicopathologic characteristics in patients with bladder cancer or the upper urinary tract.^[10,16,17]^ Similar to the findings of Shibing et al,^[8]^ UCDD was found to be an independent prognostic factor for CSS, DFS, and OS in patients with UTUC under univariate and multivariate analyses.^[8]^ In our study, UCDD remained an independent risk factor for survival outcomes on multivariate analysis. However, further studies are warranted to validate our finding in multiple centers with diverse patient populations.

Among UTUC, tumors in the renal pelvis and ureter may have different biological characteristics. Chung et al^[18]^ found that the thicker anatomic barrier of renal pelvis than ureter can lead to different consequences, and they suggested that it may be more reasonable to individually evaluate tumors in different locations. The prognosis of UTUC is strongly correlated with pathological stage, especially with invasion of the muscularis.

The gross representation of the tumor is varied according to divergent differentiations of the tumor such as papillary, nonpapillary, muscle invasive, nonmuscle invasive, presence and absence of lymphovascular invasion, presence versus absence of concurrent carcinoma in situ, etc. They were all included for microscopic evaluation. Generally, based on the size of tumor, there were at least 4 paraffin blocks of each tumor, that is, 1 cm of tumor for 1 paraffin block. For other normal part, at least 1 paraffin block is required too.

In this cohort, UCDD and higher tumor stage were independent predictor both in univariate and multivariate analysis for IRFS, CSS, and OS of ureteral tumors. In contrast, UCDD failed to be independently related to IRFS and OS in pyelocaliceal tumors. The possible reason is that diverse effect of UCDD in different location is associated with the thickness of adjacent barrier. Since the muscular layer of the ureter is much thinner than in renal pelvis, ureteric urothelial carcinoma is associated with a poorer outcome than renal pelvic urothelial carcinoma. To date, UCDD with adverse clinicopathologic characteristics such as tumor stage and grade is much more easier for cancer cells to disseminate and get aggressive in ureteral tumors than pyelocaliceal ones. If UCDD is present in ureteral tumors, its thinner muscular wall is easier for invasion. Thus, the presence of divergent differentiation, particularly in ureteral tumors, is associated with poorer survival. However, multiple-center studies are warranted to verify the relationship between the prognostic value of UCDD and tumor locations.

As it is presented in our cohort that UTUC with small cell neuroendocrine differentiation was the worst divergent subtype, all of the patients were diagnosed at an advanced stage, suffering IRFS or CSS without adjuvant chemotherapy after RNU during the follow-up. This relationship between the pathological pattern and poor prognosis was also reported by other studies for small cell neuroendocrine carcinoma differentiation of pancreas.^[19]^

In addition, history of bladder cancer, that is concomitant or previous bladder tumors, was a significantly independent predictor for IRFS in patients with UTUC on both univariate and multivariate analyses. Recently published reports of cancer survival also confirmed the similar results.^[20,21]^ In the present study, we found adjuvant chemotherapy was an important factor to improve OS in patients after RNU. Our oncological outcomes were in agreement with those of recent study series^[22]^ but not in accordance with a cohort study in Korea. Largely, this disparity may be attributed to differences in patients selection and therapeutic schedules. Besides, there is no standard chemotherapy regimens and consensus in the world. Notably, in the present study, we found that tumor necrosis was an unfavorable prognostic factor for IRFS in ureteral tumors on both univariate and multivariate analysis. This was also observed by Seisen et al.^[23]^

However, the present study has several limitations. First, the study design is retrospective and the number of cases is limited. Second, the nephroureterectomy procedures were conducted by multiple surgeons at a single tertiary care institution, introducing both the variability of intraoperative management and extent of lymph node dissection as well as a significant case selection bias. Third, the role of UCDD in lymphatic metastasis disease and the significance of Chinese populations as a predictor of worse prognosis in this disease process should be evaluated within a larger-scale investigation for further validation. Meanwhile, we included all the patients with UCDD as a single group for analysis; thus, bias owing to heterogeneity may occur. Last, the limitations of a hospital-based study cannot be ignored, which may result in the bias of patients selection.

## 5 Conclusions

In conclusion, we confirmed the imperative role of UCDD in predicting disease intraluminal recurrence and survival of patients with UTUC after receiving RNU in China. The presence of UCDD, particularly in ureteral tumors rather than pyelocaliceal tumors, is associated with poorer prognosis. As a result, more attention and follow-up should be given to patients with ureteric urothelial carcinoma.

## Acknowledgments

The authors thank the National Key Specialty Construction of Clinical Projects for the support.
